# Prediction of Germline BRCA Mutations in High-Risk Breast Cancer Patients Using Machine Learning with Multiparametric Breast MRI Features

**DOI:** 10.3390/s25175500

**Published:** 2025-09-04

**Authors:** Hyeonji Park, Kyu Ran Cho, SeungJae Lee, Doohyun Cho, Kyong Hwa Park, Yoon Sang Cho, Sung Eun Song

**Affiliations:** 1 Department of Radiology, Korea University Anam Hospital, Korea University College of Medicine, Seoul 02841, Republic of Korea; qkrguswl39@gmail.com (H.P.); krcho@korea.ac.kr (K.R.C.); 2 Dnotitia Inc., Seoul 06540, Republic of Korea; seungjae.lee@dnotitia.com (S.L.); dhcho@dnotitia.com (D.C.); 3 Department of Oncology, Korea University Anam Hospital, Korea University College of Medicine, Seoul 02841, Republic of Korea; khpark@korea.ac.kr; 4 Korea University Anam Hospital, Korea University College of Medicine, Seoul 02841, Republic of Korea; yscho187@korea.ac.kr

**Keywords:** breast cancer, magnetic resonance imaging, BRCA mutation, diagnosis, computer-assisted, machine learning

## Abstract

**Highlights:**

**What are the main findings?**

- Non-invasive machine learning model using multiparametric breast MRI (mpMRI) predicts BRCA mutations.
- Key MRI features were CAD-derived washout ≥ 19.5%, minimal/mild background parenchymal enhancement, tumor size ≥ 2.5 cm and linear discriminant analysis model achieved highest performance with AUC of 0.72 among 13 models.

**What is the implication of main findings?**

- mpMRI-based ML model enables prediction of BRCA mutations without invasive genetic testing.
- This approach provide essential insights for personalized treatment and genetic counseling.

**Abstract:**

The identification of germline BRCA1/2 (BRCA) mutations plays an important role in the treatment planning of high-risk breast cancer patients, but genetic testing may be costly or unavailable. The multiparametric breast MRI (mpMRI) features offer noninvasive imaging biomarkers that could support BRCA mutation prediction. In this study, we investigate whether mpMRI features can predict BRCA mutation status in high-risk breast cancer patients. We collected data from 231 consecutive patients (82 BRCA-positive, 149 BRCA-negative) who underwent BRCA mutation testing and preoperative MRI between 2013 and 2019. We used the mpMRI features, including computer-aided diagnosis (CAD)-derived kinetic features, morphologic features, and apparent diffusion coefficient (ADC) values from diffusion-weighted imaging (DWI). In the univariate analysis, higher CAD-derived washout component and peak enhancement, larger tumor size and angio-volume, peritumoral edema on T2-weighted imaging, axillary adenopathy, and minimal or mild background parenchymal enhancement (BPE) were significantly associated with BRCA mutation, while ADC values showed no significant differences. In the multivariate analysis, three significant predictors were washout component ≥ 19.5% (odds ratio [OR] = 3.89, *p* < 0.001), minimal or mild BPE (OR = 2.57, *p* = 0.004), and tumor size ≥ 2.5 cm (OR = 2.41, *p* = 0.004). Using these predictors, we compared the predictive performance of 13 ML models through 30 repeated runs and achieved the highest performance (AUC = 0.72). In conclusion, ML models integrating mpMRI features demonstrated good performance for predicting BRCA mutations in high-risk patients. This noninvasive approach may aid personalized treatment planning and genetic counseling.

## 1. Introduction

Hereditary breast cancer accounts for 5–10% of breast cancer cases [1,2,3], with approximately 16% of these related to germline Breast Cancer Gene (BRCA) 1 or 2 mutations. According to a prospective study, the risk of developing breast cancer by age 80 years is 72% for those with a BRCA1 mutation and 69% for those with a BRCA2 mutation. Therefore, genetic testing to identify BRCA mutations in patients with high risk (a significant risk ≥ 10% of mutation) is commonly employed [1,2,3,4,5,6] and recommended for breast cancer patients ≤ 65 years, patients with a family history of breast or ovarian cancer, early-onset triple-negative breast cancer, and bilateral breast cancer [7,8,9]. The presence of BRCA mutations guides the use of DNA-damaging agents (e.g., platinum-based chemotherapy) and targeted therapies, such as poly ADP-ribose polymerase inhibitors (PARPi). These strategies exploit the underlying homologous recombination deficiency in BRCA-mutated cancers, leading to better clinical outcomes compared to standard treatments. Indeed, recent studies proved a substantial benefit of PARPi for progression-free survival in BRCA-mutated cancer patients [10,11]. Accordingly, the significance of identifying BRCA mutations is becoming increasingly emphasized for treatment planning.

Based on studies demonstrating MRI’s superior sensitivity in cancer detection compared to mammography or sonography, most guidelines, such as those from the American Cancer Society, currently recommend an annual breast MRI in addition to mammography for individuals with BRCA mutations [12,13,14,15]. Several studies employing breast MRI have demonstrated a significant correlation between morphologic MRI features of breast cancers and BRCA mutation status [16,17,18,19]. In these studies, breast cancers in BRCA mutation carriers tend to exhibit a higher prevalence of round shape, circumscribed margins, rim enhancement, and posterior location [16,17,18,19], and show high T2 signal intensity, which is characteristic of benign lesions. Specifically, breast cancers in patients with BRCA1 mutations tend to exhibit a benign appearance and display more circumscribed margins and rim enhancement compared to those with BRCA2 mutations [13,18,19]. From breast MRI, we can extract not only qualitative morphologic features but also obtain quantitative kinetic features derived from computer-aided diagnosis (CAD), and apparent diffusion coefficient (ADC) values from diffusion-weighted images (DWI). Regarding kinetic features, one study that assessed quantitative kinetic analysis in patients with high risk for breast cancer revealed that vascular properties of background parenchymal enhancement (BPE) were different between BRCA carriers and noncarriers [20]. Another study using qualitative kinetic features insisted that there was no significant difference in kinetics between BRCA-positive and BRCA-negative groups [21]. Regarding ADC values from DWI, it was known to correlate with prognostic biomarkers in breast cancer, including hormone receptor status and proliferation marker Ki-67. However, previous studies have not utilized ADC value as a parameter for BRCA prediction. Therefore, we incorporated ADC value into our analysis.

Recently, prediction models based on machine learning (ML) algorithms using mpMRI features have emerged as an economical and non-invasive approach for precision medicine [22,23]. Regarding BRCA prediction, one recent study used MRI texture features after manual segmentation of breast cancers from 41 patients and revealed that the prediction model using texture features achieved the highest predictive value with an area under the curve (AUC) of 0.86 for BRCA prediction [24]. However, to the best of our knowledge, there has been no relevant literature using multiparametric MRI (mpMRI) features integrating morphologic, CAD-assessed kinetic features, and ADC values for constructing prediction models for BRCA mutation.

Therefore, the purpose of this study was to investigate whether ML-based prediction models using mpMRI features could predict BRCA mutations in breast cancer patients fulfilling high-risk criteria.

## 2. Materials and Methods

### 2.1. Patients

**Figure 1:**
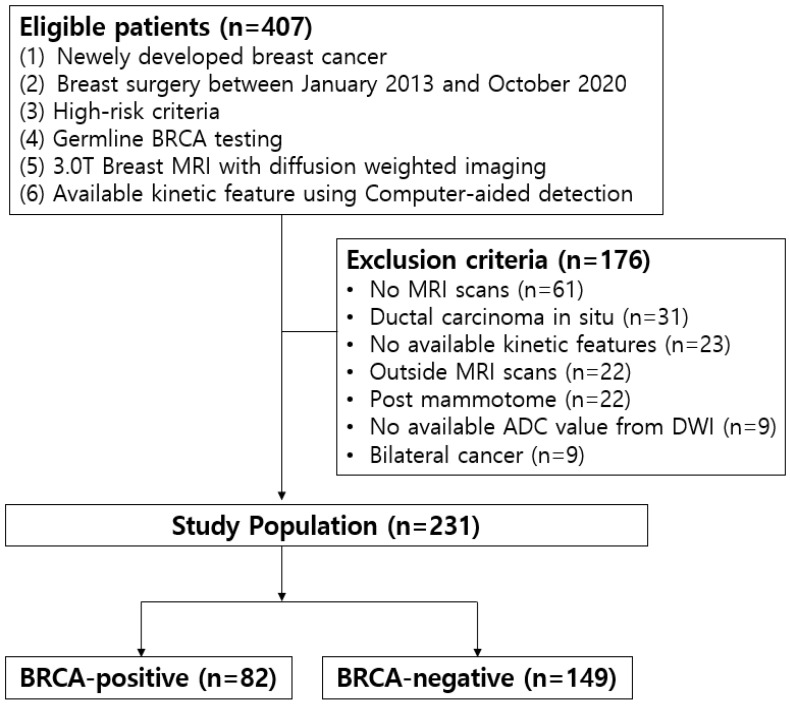
Study population.

This study received approval from our institutional review board (IRB). The requirement for written, informed patient consent was waived by the IRB (IRB number 2023AN0368). Our institution’s medical records from January 2013 to October 2019 were retrospectively reviewed. From this review, 407 consecutive breast cancer patients tested for germline BRCA mutation due to high-risk criteria (breast cancer patients ≤ 65 years, patients with a family history of breast or ovarian cancer, early-onset triple-negative breast cancer, and bilateral breast cancer) were collected. Our inclusion criteria were as follows: newly diagnosed invasive breast cancer through core needle biopsy, underwent a preoperative MRI with available kinetic features and ADC values using a CAD, and received breast cancer surgery in our institution. Among them, 176 patients were excluded from the study due to the following reasons: no MRI scans (*n* = 61), ductal carcinoma in situ (*n* = 31), outside breast MRI scans (*n* = 22), no available kinetic features or ADC values with our CAD system (*n* = 32), mammotome excision or biopsy before MRI scans (*n* = 22), and diagnosis of bilateral cancer (*n* = 9) because it was not possible to determine which side’s cancer was associated with the BRCA mutation. Finally, 231 women (mean age ± standard deviation, 50.3 years ± 12.6; age range, 26–87 years) were enrolled (Figure 1). Among them, 82 patients were identified as having a BRCA mutation (BRCA-positive) while the remaining 149 were wild type (BRCA-negative).

### 2.2. MRI Examination

Bilateral breast MRI examinations were performed on a 3.0T scanner (Achieva 3.0TTX; Philips Healthcare, Best, The Netherlands) using a specialized seven-channel breast array coil in the axial orientation. All patients underwent MRI scans in the prone position. Both sides of fat-suppressed T2-weighted images (T2WI) in the axial plane were obtained (repetition time ms/echo time ms, 5375/65; flip angle, 90°, matrix, 620 × 303; field of view, 340 × 340 mm^2^; section thickness, 3 mm; section gap, 0 mm). DWI with fat suppression was acquired using an echo-planar-imaging sequence in the axial orientation (5417/72; flip angle, 90°, matrix, 96 × 126; field of view, 320 × 320 mm^2^; section thickness, 3 mm; section gap, 0 mm; b values, 0 and 1000 s/mm^2^). Utilizing a three-dimensional fat-suppressed T1-weighted fast spoiled gradient-echo sequence (5/2; matrix, 436 × 436; flip angle, 12°; field of view, 340 × 340 mm^2^; section thickness, 1 mm; no gap), a total of six dynamic sequences comprising one pre-contrast and the other post-contrast were acquired following an intravenous bolus injection of gadoterate at a dose of 0.1 mmol/kg (Dotarem; Guerbet, Villepinte, France).

### 2.3. MRI Image Analysis

**Figure 2:**
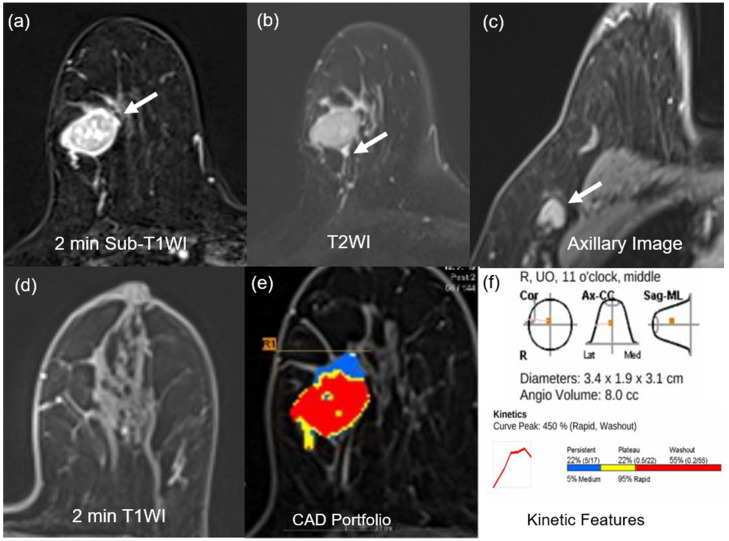
MR images of a 75-year-old woman with a 3.4-cm surgically verified invasive ductal breast cancer and germline BRCA-positivity (**a**) Axial contrast-enhanced T1-weighted 2 min subtraction image shows an oval, circumscribed, rim-enhancing mass in the right breast (arrow). (**b**) The T2-weighted image shows peritumoral edema near the mass (arrow). (**c**) Axillary image shows an axillary adenopathy (arrow). (**d**) Axial contrast-enhanced T1-weighted 2 min after contrast enhancement image shows minimal background parenchymal enhancement. (**e**,**f**) An auto-portfolio of the CAD system demonstrated 3.4 cm in the largest tumor size and 8.0 cc in angio-volume above the enhancement threshold. Delayed-washout component was 55%.

**Figure 3:**
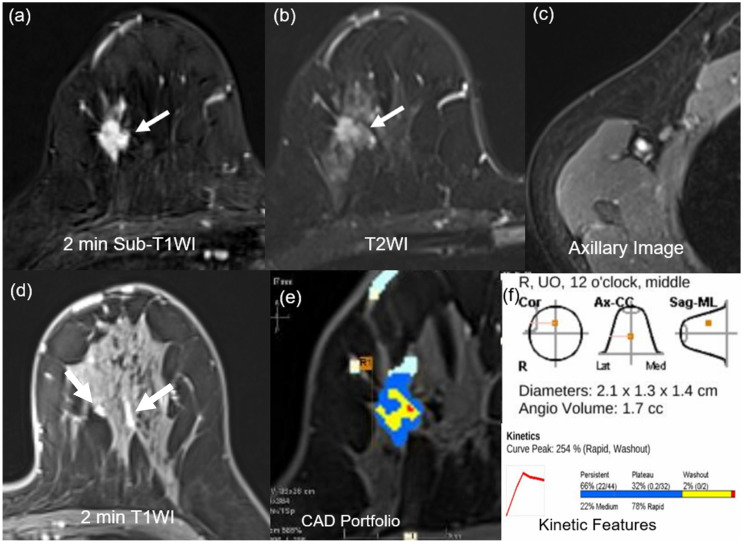
MR images of a 40-year-old woman with a 2.1-cm surgically verified invasive ductal breast cancer and germline BRCA-negativity (**a**) Axial contrast-enhanced T1-weighted 2 min subtraction image shows an irregular, spiculated, heterogeneously enhancing mass in the right breast (arrow). (**b**) The T2-weighted image shows no peritumoral edema near the mass (arrow). (**c**) Axillary image shows no axillary adenopathy. (**d**) Axial contrast-enhanced T1-weighted 2 min after contrast enhancement image shows moderate background parenchymal enhancement (arrows). (**e**,**f**) An auto-portfolio of the CAD system demonstrated 2.1 cm in the largest tumor size and 1.7 cc in angio-volume above the enhancement threshold. Delayed-washout component was 2%.

For morphologic features, two breast radiologists (18 and 9 years of experience) evaluated breast MRI findings in consensus, according to the 2013 Breast Imaging Reporting and Data System MR lexicon [25]. The assessed MRI morphologic features included the extent of fibroglandular tissue (FGT) and BPE, and determination of whether the lesion exhibited mass or non-mass enhancement (NME). For the masses, the assessment of their shape, margin, and internal enhancement pattern was evaluated (Figure 2 and Figure 3). On T2WI, intra-tumoral high signal intensity, indicated visually by a signal higher than that of blood vessels, water, or surrounding parenchymal tissues, as well as peritumoral edema, defined by high SI surrounding the tumor, were also assessed [25,26,27]. In patients with multiple lesions, the largest lesion was evaluated. The presence of axillary adenopathy was determined if meeting one or more of the following criteria: abnormal shape and margin of the lymph node, abnormal cortical thickness, or effacement of the lymph node fatty hilum [25].

### 2.4. Kinetic Feature Analysis

For the kinetic feature, one breast radiologist used a CAD system (CAD stream, version 6.0, Confirma, Kirkland, WA, USA). According to the report, suggesting the most suitable enhancement threshold for the CAD system to be between 50% and 60% [28], a 50% threshold was selected for defining enhancement. Using this threshold, all enhancing lesions were colored to generate an angio-map. On the angio-map, the largest tumor was identified by a radiologist, and the peak enhancement, angio-volume, tumor size, as well as the enhancement profile for the early and delayed phases, were evaluated. The early phase enhancement profile was classified into two types: medium or rapid enhancement, while the delayed phase enhancement profile was classified into three types: persistent, plateau, or washout.

### 2.5. DWI Analysis

As well as kinetic features, a CAD system processed all DWI images. The ADC map consisted of b-values 0 and 1000 s/mm^2^. The regions of interest (ROIs) of each lesion were independently reviewed and delineated manually by one radiologist. The largest cross-section of the entire tumor was measured twice, with reference to T2WI and angio-map, and while avoiding cystic lesions. The ADC values for each ROI were measured, including the minimum, median, and maximum.

### 2.6. Histopathologic Data Analysis

We acquired surgically excised tumor tissue and employed the immunohistochemical (IHC) technique with labeled streptavidin biotin to confirm estrogen receptor (ER) and progesterone receptor (PR) positivity, defined as positive staining in 1% or more of the nuclei in ten high-power fields [29]. HER2 negativity was determined not only by an IHC score of 0 or 1+ but also by an IHC score of 2+ without HER2 gene amplification detected through fluorescence in situ hybridization. Molecular subtypes were categorized as follows: luminal (ER or PR-positive, HER2-negative, regardless of Ki-67), HER2-enriched (HER2-positive, regardless of ER or PR status), and triple-negative (ER, PR, HER2-negative).

### 2.7. Statistical Analysis

Statistical analysis was conducted to compare the mpMRI features, such as morphological, kinetic features, and ADC values, between BRCA-positive and BRCA-negative groups. The x2 test or Fisher’s exact test was applied to categorical variables, while the Student *t*-test was applied to normally distributed continuous variables, and the Mann–Whitney U test to non-normally distributed continuous variables. To obtain the optimal cutoff values of kinetic features and ADC values for predicting BRCA positivity, receiver operating characteristic (ROC) curve analysis was performed by using the maximum Youden index (i.e., sensitivity + specificity − 1).

For the selection of the mpMRI feature significantly associated with BRCA mutation, logistic regression analysis was used. Covariates with *p*-values < 0.05 in the univariate analysis were selected and used for multivariate analysis. Statistical analyses were conducted utilizing SPSS version 20.0 for Windows (SPSS Inc., Chicago, IL, USA), as well as open-source R software (version 3.5.1; R Foundation for Statistical Computing, Vienna, Austria), and Python (Python Software Foundation, version 3.7.4).

## 3. Results

### 3.1. Patient Characteristics

Among the 231 patients, 82 (35.5%) were in the BRCA-positive group and 149 (64.5%) were in the BRCA-negative group. Table 1 presents the results of the clinicopathologic features and distribution of the molecular subtypes according to the BRCA mutation. As compared to the BRCA-negative group, the BRCA-positive group showed higher mean age (55.0 ± 12.2 vs. 47.8 ± 12.1, *p* < 0.001), higher histologic grade of 3 (52.4% vs. 32.2%, *p* = 0.003), and larger invasive tumor size (2.8 ± 2.1 vs. 2.2 ± 1.9 cm, *p* = 0.039). In terms of axillary lymph node metastasis and lymphovascular invasion, there were no statistical significances (*p* > 0.05). Regarding molecular subtypes, the BRCA-positive group had more triple-negative subtype (20.7%) than the BRCA-negative group (12.1%), while the BRCA-negative group had more luminal subtype (64.4%) than the BRCA-positive group (47.6%).

### 3.2. MRI Morphological Features According to BRCA Mutation

When comparing BRCA-positive and BRCA-negative groups, significant results were observed in BPE, peritumoral edema on T2WI, and axillary adenopathy among the various MRI morphologic features. The BRCA-positive group showed a significantly greater number of minimal or mild BPE (76.8% vs. 55.7%, *p* = 0.001), peritumoral edema on T2WI (73.2% vs. 59.1%, *p* = 0.022), and axillary adenopathy (70.7% vs. 56.4%, *p* = 0.035) (Table 2).

### 3.3. Kinetic Features and ADC Values According to BRCA Mutation

Among kinetic features, mean tumor size and angio-volume were significantly larger in the BRCA-positive group than in the BRCA-negative group (3.5 ± 1.0 cm vs. 2.6 ± 1.7 cm, *p* < 0.001 for tumor size; 16.1 ± 35.2 cm^3^ vs. 6.6 ± 12.4 cm^3^, *p* < 0.001 for angio-volume). In addition, tumors of the BRCA-positive group showed higher peak enhancement (417.2 ± 306.6% vs. 302.5 ± 193.8%, *p* = 0.001) and higher washout component (31.9 ± 21.6% vs. 19.8 ± 19.9%, *p* < 0.001). However, the analysis of ADC values did not show any significant differences (Table 2).

### 3.4. Multiparametric Features Associated with the Germline BRCA-Positive Group

Univariate logistic regression analysis was conducted to confirm the association between mpMRI features and BRCA mutation. At univariate analysis, minimal or mild BPE, peritumoral edema, axillary adenopathy, tumor size ≥ 2.5 cm, angio-volume ≥ 3.9 cm^3^, peak enhancement ≥ 314.5%, delayed phase-persistent component < 32.5%, and delayed phase-washout component ≥ 19.5% were associated with BRCA mutation. Multivariate analysis showed that delayed phase-washout component ≥ 19.5% (odds ratio [OR] = 3.89; *p* < 0.001), minimal to mild BPE (OR = 2.57; *p* = 0.004), and tumor size ≥ 2.5 cm (OR = 2.41; *p* = 0.004) were respectively associated with BRCA mutation (Table 3, Figure 2 and Figure 3).

### 3.5. Diagnostic Performance of Prediction Models

We performed predictive analysis to investigate the effectiveness of significant features selected from Table 1, Table 2 and Table 3 (multivariate). In this study, we compared a total of thirteen ML models, including Adaboost (AB), CatBoost (CB), Decision Tree (DT), Logistic Regression (LR), Random Forest (RF), Support Vector Machine (SVM), k-NN, Naive Bayes (NB), multilayer perceptron (MLP), linear discriminant analysis (LDA), quadratic discriminant analysis (QDA), Extra Trees (ET), and Elastic Net (EN). All codes and implementations have been made publicly available at https://github.com/YoonSangCho/MultiparametricMRI (accessed on 22 August 2025).

In our experiments, for the data preprocessing, we first randomly split the data into training (80%) and testing (20%) datasets based on a random seed. We then performed Z-score normalization by fitting the scaler on the training data and transforming both the training and testing data. During model training, we performed a grid search to select the best hyperparameters of the model based on 5-fold stratified cross-validation on the training set, yielding the highest AUC score. For the grid search, we specifically included hyperparameters that address class imbalance, such as class weights or imbalanced learning algorithms, to ensure the model’s robustness. After selecting the best hyperparameters, we retrained the model on the training set and evaluated it on the held-out test set. We repeated the experiments 30 times with different random seeds using the above models to ensure the robustness and reproducibility of the results.

**Figure 4:**
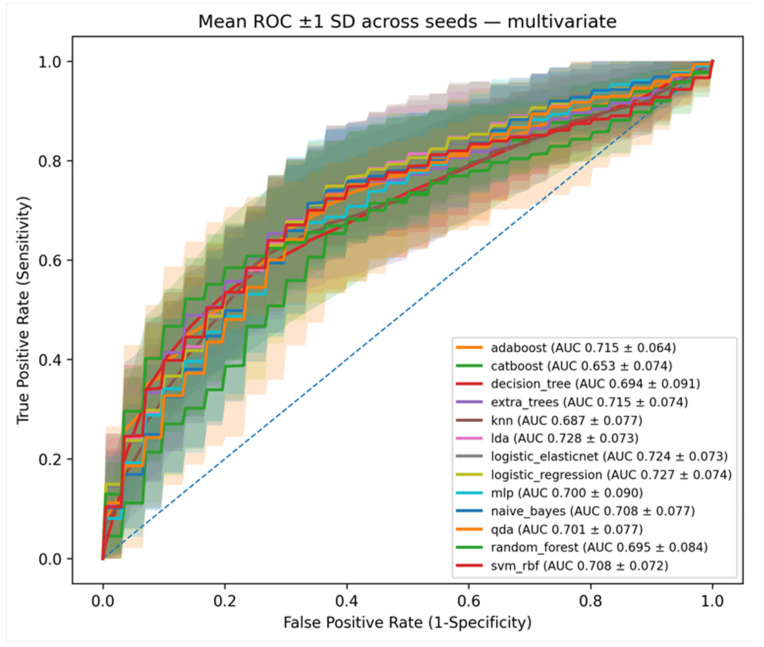
Graphs show areas under the receiver operating characteristic curve (AUCs) of machine learning models using significant mpMRI features (Morphologic-Kinetic) acquired from multivariate analysis. The solid line represents the mean AUC, and the shaded area indicates the standard deviation.

Table 4 show the predictive results for each model and feature set. Baseline models using single feature groups (clinical, kinetic, or morphologic alone) showed modest performance, with AUC values ranging from 0.64 to 0.70. By contrast, integrating multiple feature groups consistently improved predictive accuracy. The highest overall performance was achieved when combining clinical and kinetic features (AUC = 0.77 ± 0.06), followed closely by the combination of clinical and mpMRI features (AUC = 0.77 ± 0.06). These findings suggest that selecting informative variables from complementary feature domains yields higher efficiency without compromising accuracy (Figure 4).

Moreover, the multivariate model using the mpMRI feature set alone achieved an AUC of 0.72, demonstrating the value of combining complementary imaging features even in reduced settings. Importantly, when using only the three significant features identified by multivariate logistic regression analysis, the models still maintained robust performance around 0.72, underscoring the effectiveness of careful feature selection.

Overall, these results confirm that multiparametric MRI-derived features substantially enhance predictive performance compared with baseline models relying only on clinical or pathological information.

## 4. Discussion

Our study demonstrated that CAD-assessed kinetic features, such as higher washout component and larger tumor size, and morphologic features representing minimal or mild BPE, are independent predictors of BRCA mutation. The baseline model using only clinical or morphologic or kinetic features showed limited predictive performance, whereas our clinical-mpMRI based approach achieved substantially improved results with AUC values of approximately 0.77 across 13 ML classifiers. Even when restricted to three mpMRI features without clinical features, the models maintained robust performance with AUC values around 0.72, confirming the stability of our approach. Established BRCAPRO and BOADICEA are risk prediction software/web tools that are generally available free of charge or provided to genetic counselors and healthcare providers and these tools also have demonstrated AUCs ranging from 0.72 to 0.80. Therefore, our prediction model using mpMRI features demonstrates AUC comparable to that of BRCAPRO and BOADICEA.

Among various mpMRI features, the CAD-assessed washout component was the most significant predictor for BRCA mutation. One study noted that 81.5% of cancers with BRCA mutation showed a washout curve, but there was no significant difference in kinetics between BRCA-positive and BRCA-negative groups [21]. The study mentioned above evaluated the kinetic curve through radiologists’ visual assessment, whereas our study utilized CAD-assessed quantitative analysis, which represents a significant distinction. Since a CAD-assessed higher washout component was a well-known factor significantly associated with poorer disease-free survival [30], it can be inferred that a higher washout component in BRCA mutation carriers may be linked to a worse prognosis.

The second most important mpMRI feature for predicting BRCA mutation was CAD-assessed larger tumor size. In our study, tumors in BRCA-positive patients have a larger mean pathologic tumor size and a higher prevalence of triple-negative breast cancer, which could explain the significant differences observed in CAD-assessed tumor size. Our data was not collected based on tumor size, but rather consecutively gathered from high-risk groups eligible for BRCA testing, making this result interesting. One study that investigated the association between quantitative MRI features and breast cancer subtypes also revealed that larger tumor size is predictive of more aggressive cancer phenotypes, which are often associated with BRCA mutations [30].

An interesting finding in our study is that minimal to mild BPE aids in predicting BRCA mutations. Our result was similar to those of studies where the BPE in BRCA mutation carriers was found to be lower compared to the control group [20,31]. Grubstein et al. [31] insisted that BRCA mutation carriers demonstrated lower levels of BPE and FGT than age-matched non-carriers. These differences are likely influenced by hormonal status and defective DNA damage repair because BRCA mutation carriers are thought to have an increased risk contributing to carcinogenesis through the loss of BRCA gene function, which mediates DNA repair, replication, and RNA processing for suppression of DNA [32].

Recently, ML has been utilized to develop prediction models. Several studies on breast cancer have claimed that prediction models based on ML and utilizing various mpMRI features can predict the outcomes of neoadjuvant chemotherapy, the probability of recurrence or axillary lymph node metastasis, the level of Ki-67, and histological grade in luminal cancer [20,21,33,34]. Similar to these studies, our study employed 13 ML models to identify the most appropriate model for accurate prediction. In the clinical flow, the ML model can be successfully used as a non-invasive, point-of-care decision-support tool. Specifically, after a patient undergoes a standard mpMRI scan for high-risk breast cancer screening, the CAD-derived kinetic features (e.g., washout component or tumor size), and morphologic variables (e.g., BPE) are collected. All these features can then be input into the trained ML model, which provides a probability score for BRCA mutation. This prediction can complement genetic counseling to guide the prioritization of genetic testing for high-risk individuals, and support personalized patient management decisions, all without the need for additional invasive procedures.

There are several limitations to our study. First, this study was designed retrospectively and conducted at a single tertiary academic facility. Second, the absence of data on demographic factors and personal histories, such as menstrual cycle and the presence of other tumors related to BRCA mutation, may have led to an oversight in considering their potential impact on the mpMRI features in our analysis. Third, we did not separately compare BRCA1 and BRCA2 mutations because of the small number of patients with BRCA mutations; it is possible that we failed to capture the distinctive mpMRI features of BRCA1 and BRCA2 or obtained offsetting results. Finally, in this study, we demonstrated performance through repeated internal validation. The generalizability of our findings needs to be confirmed by future prospective studies with a larger external patient cohort.

## 5. Conclusions

In conclusion, CAD-assessed higher values of the washout kinetic component and larger tumor size, and lower levels of BPE were associated with BRCA mutation. The results of our study aid in understanding the relationship between breast cancer with BRCA mutation and mpMRI features. While these results may not be fully sufficient for a standalone diagnostic tool, the model holds value as a pre-screening strategy to identify patients at higher risk of carrying BRCA mutations, thereby guiding more targeted and cost-effective genetic testing.

## Figures and Tables

**Table 1:** Clinicopathologic features and molecular subtypes according to the BRCA mutation.

Features	All (*n* = 231)	BRCA-Positive (*n* = 82)	BRCA-Negative (*n* = 149)	*p* Value
Patients’ Mean age (years)	50.3 ± 12.6	55.0 ± 12.2	47.8 ± 12.1	<0.001
Histologic Grade				0.003
1 or 2	140 (60.6)	39 (47.6)	101 (67.8)	
3	91 (39.4)	43 (52.4)	58 (32.2)	
Mean invasive tumor size (cm)	2.4 ± 2.0	2.8 ± 2.1	2.2 ± 1.9	0.039
Axillary lymph node metastasis	86 (37.2)	34 (41.5)	52 (34.9)	0.394
Lymphovascular invasion	43 (18.6)	18 (22.0)	25 (16.8)	0.378
Molecular Subtypes				0.005
Luminal	135	39 (47.6)	96 (64.4)	
HER-2	61	26 (31.7)	35 (23.5)	
Triple-Negative	35	17 (20.7)	18 (12.1)	

**Table 2:** Multiparametric MRI features according to BRCA mutation.

Features	All (*n* = 231)	BRCA-Positive (*n* = 82)	BRCA-Negative (*n* = 149)	*p* Value
**Morphologic features**				
Amount of fibroglandular tissue				0.201
Nondense	48	20 (24.4)	28 (18.8)	
Dense	183	62 (75.6)	121 (81.2)	
Background parenchymal enhancement				0.001
Minimal or mild	146	63 (76.8)	83 (55.7)	
Moderate or marked	85	19 (23.2)	66 (44.3)	
Lesion type				1.000
Mass *	212	75 (91.5)	137 (91.9)	
Non-mass enhancement	19	7 (8.5)	12 (8.1)	
Mass shape *				0.513
Round to oval	69	24 (32.0)	45 (32.8)	
Irregular	143	51 (68.0)	92 (67.2)	
Mass margin *				0.299
Circumscribed	29	12 (16.0)	17 (12.4)	
Not circumscribed	183	63 (84.0)	120 (87.6)	
Mass internal enhancement *				0.079
Homo- or heterogeneous	163	53 (70.7)	110 (80.3)	
Rim	49	22 (29.3)	27 (19.7)	
Intratumoral high SI on T2WI				0.494
No	103	36 (43.9)	67 (45.0)	
Yes	128	46 (56.1)	82 (55.0)	
Peritumoral edema on T2WI				0.022
Absent	83	22 (26.8)	61 (40.9)	
Present	148	60 (73.2)	88 (59.1)	
Axillary lymph node enlargement				0.035
Absent	89	24 (29.3)	65 (43.6)	
Present	142	58 (70.7)	84 (56.4)	
**Kinetic features on CAD**				
Tumor size (cm) ^†^	3.0 ± 1.9	3.5 ± 1.0	2.6 ± 1.7	<0.001
Angio-volume (cm^3^) ^†^	9.9 ± 23.6	16.1 ± 35.2	6.6 ± 12.4	<0.001
Peak enhancement (%) ^†^	343.2 ± 245.5	417.2 ± 306.6	302.5 ± 193.8	0.001
Early phase-medium component (%) ^†^	29.8 ± 34.3	25.3 ± 33.1	32.4 ± 34.7	0.252
Early phase-rapid component (%) ^†^	70.1 ± 34.2	74.8 ± 33.1	67.5 ± 34.8	0.242
Delayed phase-persistent component (%)	43.5 ± 25.2	36.1 ± 21.6	47.6 ± 26.1	0.002
Delayed phase-plateau component (%) ^†^	32.3 ± 14.5	31.9 ± 11.6	32.6 ± 16.2	0.673
Delayed phase-washout component (%) ^†^	24.1 ± 21.3	31.9 ± 21.6	19.8 ± 19.9	<0.001
**ADC values on DWI**				
Mean ADC ^†^ (×10^−3^ mm^2^/s)	1.00 ± 0.21	0.97 ± 0.12	1.02 ± 0.25	0.145
Minimum ADC ^†^ (×10^−3^ mm^2^/s)	0.72 ± 0.43	0.78 ± 0.40	0.69 ± 0.44	0.096
Maximum ADC ^†^ (×10^−3^ mm^2^/s)	1.43 ± 0.52	1.45 ± 0.52	1.42 ± 0.52	0.784
SD of ADC ^†^ (×10^−3^ mm^2^/s)	0.18 ± 0.08	0.05 ± 0.29	0.02 ± 0.10	0.055

Data are numbers of patients, with percentages in parentheses. * Mass shape, margin, and internal enhancement were calculated with a denominator of 212 masses. ^†^ Numbers are means ± standard deviations. *T2WI* = T2-weighted image. *CAD* = computer-aided diagnosis. *ADC* = apparent diffusion coefficient. *DWI* = diffusion weighted imaging. *SD* = standard deviation.

**Table 3:** Univariate and multivariate logistic regression analysis for multiparametric MRI features.

Features	Univariate Analysis		Multivariate Analysis	
	Odds Ratio	*p* Value	Adjusted Odds Ratio	*p* Value
**Morphologic features**				
Background parenchymal enhancement		0.002		0.004
Minimal or mild	2.64 (1.44–4.83)		2.57 (1.34–4.93)	
Moderate or marked	Reference		Reference	
Peritumoral edema		0.034		
Yes	1.89 (1.05–3.40)			
No	Reference			
Axillary lymph node enlargement		0.033		
Yes	1.87 (1.05–3.32)			
No	Reference			
**Kinetic Features**				
CAD-derived Tumor size (cm)		0.002		0.004
≥2.5	2.44 (1.40–4.24)		2.41 (1.32–4.38)	
<2.5	Reference		Reference	
CAD-derived Angio-volume (cm^3^)		0.001		
≥3.9	2.51 (1.44–4.36)			
<3.9	Reference			
CAD-derived Peak Enhancement (%)		<0.001		
≥314.5	2.88 (1.65–5.04)			
<314.5	Reference			
Delayed phase-persistent component (%)		0.020		
<32.5	1.92 (1.10–3.32)			
≥32.5	Reference			
Delayed phase-washout component (%)		<0.001		<0.001
≥19.5	4.28 (2.37–7.71)		3.89 (2.11–7.15)	
<19.5	Reference		Reference	

Numbers in parentheses are 95% confidence intervals.

**Table 4:** Diagnostic performance of ML models and feature sets.

	Univariate	Univariate	Univariate	Univariate	Univariate	Univariate	Multivariate	Univariate	Multivariate
	Clinical	Kinetic	Morphologic	Clinical + Kinetic	Clinical + Morphologic	Clinical + mpMRI(Morphologic + Kinetic)	Clinical + mpMRI(Morphologic + Kinetic)	mpMRI (Morphologic + Kinetic)	mpMRI (Morphologic + Kinetic)
**AB**	**0.6916 (0.0683)**	0.6687 (0.0763)	0.6370 (0.0702)	0.7616 (0.0631)	0.6844 (0.0731)	**0.7627 (0.0644)**	0.7654 (0.0647)	0.6953 (0.0684)	0.7147 (0.0654)
**CB**	0.6041 (0.0785)	0.5895 (0.0893)	0.6200 (0.0674)	0.7221 (0.0689)	0.6208 (0.0708)	0.7288 (0.0718)	0.7229 (0.0581)	0.6404 (0.0817)	0.6531 (0.0754)
**DT**	0.6487 (0.0853)	0.6528 (0.0756)	0.6195 (0.0673)	0.7396 (0.0597)	0.6882 (0.0799)	0.7344 (0.0659)	0.7456 (0.0675)	0.6925 (0.0848)	0.6937 (0.0926)
**ET**	0.6750 (0.0598)	**0.7039 (0.0831)**	0.6177 (0.0709)	0.7395 (0.0686)	0.6873 (0.0651)	0.7306 (0.0700)	0.7274 (0.0687)	0.7088 (0.0776)	0.7149 (0.0750)
**KNN**	0.6066 (0.0839)	0.6665 (0.0916)	0.6009 (0.0805)	0.7065 (0.0753)	0.6619 (0.0585)	0.6996 (0.0735)	0.7285 (0.0715)	0.6635 (0.0670)	0.6866 (0.0783)
**LDA**	0.6849 (0.0630)	0.7009 (0.0823)	0.6430 (0.0698)	0.7692 (0.0659)	0.6932 (0.0606)	0.7588 (0.0689)	0.7541 (0.0670)	**0.7196 (0.0781)**	**0.7277 (0.0742)**
**EN**	0.6899 (0.0643)	0.6929 (0.0815)	0.6403 (0.0711)	**0.7725 (0.0635)**	0.6830 (0.0565)	0.7586 (0.0628)	0.7676 (0.0675)	0.7133 (0.0768)	0.7245 (0.0744)
**LR**	0.6893 (0.0637)	0.6994 (0.0830)	**0.6422 (0.0706)**	0.7701 (0.0668)	0.6990 (0.0606)	0.7530 (0.0680)	0.7653 (0.0681)	0.7173 (0.0804)	0.7268 (0.0750)
**MLP**	0.6392 (0.0979)	0.6821 (0.0910)	0.6115 (0.0769)	0.7250 (0.0718)	0.6731 (0.0795)	0.7335 (0.0713)	0.7169 (0.0850)	0.7078 (0.0721)	0.7001 (0.0912)
**NB**	0.6555 (0.0754)	0.6959 (0.0838)	0.6346 (0.0714)	0.7253 (0.0760)	0.6679 (0.0764)	0.7300 (0.0764)	0.7141 (0.0812)	0.7087 (0.0820)	0.7075 (0.0786)
**QDA**	0.6495 (0.0757)	0.6820 (0.0745)	0.6332 (0.0735)	0.7252 (0.0637)	0.6435 (0.0701)	0.7157 (0.0665)	0.7285 (0.0689)	0.6749 (0.0650)	0.7013 (0.0785)
**RF**	0.6816 (0.0760)	0.6464 (0.0867)	0.6185 (0.0716)	0.7538 (0.0635)	**0.7082 (0.0781)**	0.7466 (0.0650)	0.7669 (0.0621)	0.6788 (0.0918)	0.6954 (0.0858)
**SVM**	0.6230 (0.0950)	0.6783 (0.0798)	0.5984 (0.1132)	0.7664 (0.0630)	0.6637 (0.1113)	0.7470 (0.0670)	**0.7724 (0.0680)**	0.7052 (0.0722)	0.7076 (0.0733)

The results are presented as mean AUC (standard deviation). The best results are marked in bold and underlined for each feature set.

## Data Availability

The raw data supporting the conclusions of this article will be made available by the authors on request.
